# A novel laser desorption/ionization method using through hole porous alumina membranes

**DOI:** 10.1002/rcm.8252

**Published:** 2018-09-27

**Authors:** Yasuhide Naito, Masahiro Kotani, Takayuki Ohmura

**Affiliations:** ^1^ The Graduate School for the Creation of New Photonics Industries 1955‐1 Kurematsu‐cho, Nishi‐ku Hamamatsu 431‐1202 Japan; ^2^ Hamamatsu Photonics K.K. 314‐5 Shimokanzo Iwata Japan

## Abstract

**Rationale:**

A novel matrix‐free laser desorption/ionization method based on porous alumina membranes was developed. The porous alumina membranes have a two‐dimensional (2D) ordered structure consisting of closely aligned straight through holes of sub‐micron in diameter that are amenable to mass production by industrial fabrication processes.

**Methods:**

Considering a balance between the ion generating efficiency and the mechanical strength of the membranes, the typical values for the hole diameter, open aperture ratio and membrane thickness were set to 200 nm, 50% and 5 μm, respectively. The membranes were coated with platinum on a single side that was exposed to the laser. Evaluation experiments were conducted on the feasibility of this membrane structure for an ionization method using a single peptide and mixed peptides and polyethylene glycol samples and a commercial matrix‐assisted laser desorption/ionization (MALDI) time‐of‐flight mass spectrometer in the positive ion mode.

**Results:**

Results showed a softness of ionization and no sweet spot nature. The capillary action of the through holes with very high aspect ratio enables several loading protocols including sample impregnation from the surface opposite to the laser exposure side.

**Conclusions:**

The feasibility study indicates that the through hole porous alumina membranes have several advantages in terms of usefulness over the conventional surface‐assisted laser desorption ionization (SALDI) methods. The proposed novel ionization method is termed Desorption Ionization Using Through Hole Alumina Membrane (DIUTHAME).

## 1 INTRODUCTION

Surface‐assisted laser desorption/ionization (SALDI) methods, which utilize the optophysical properties of particular solid structures instead of the spectral properties of chemical compounds, can be implemented on existing matrix‐assisted laser desorption/ionization (MALDI) mass spectrometers, and thus have relevance as a complementary technology to MALDI even if not replacing it. Particularly, they would be effective approaches for analytes in the low‐*m/z* region that suffer from intense peaks related to matrices in MALDI.

To date a number of ionization techniques that belong to SALDI have been proposed,^1^, ^2^, ^3^, ^4^, ^5^, ^6^, ^7^, ^8^, ^9^, ^10^, ^11^, ^12^, ^13^, ^14^, ^15^, ^16^, ^17^, ^18^, ^19^, ^20^, ^21^, ^22^, ^23^, ^24^, ^25^, ^26^, ^27^, ^28^, ^29^, ^30^, ^31^, ^32^, ^33^, ^34^, ^35^, ^36^, ^37^, ^38^, ^39^, ^40^, ^41^, ^42^, ^43^, ^44^, ^45^, ^46^, ^47^, ^48^, ^49^, ^50^, ^51^, ^52^, ^53^, ^54^, ^55^, ^56^, ^57^, ^58^, ^59^, ^60^, ^61^, ^62^, ^63^, ^64^, ^65^, ^66^, ^67^, ^68^, ^69^, ^70^, ^71^, ^72^, ^73^, ^74^, ^75^, ^76^, ^77^, ^78^, ^79^, ^80^, ^81^ including commercialized devices.^46^, ^76^, ^78^ They can be categorized into two groups.

The techniques in the first category realize mixing and/or interactions of analytes and fine particulate solids, e.g. nanoparticles as typical, in treatable forms such as suspension liquids in order to ensure that the photon energies of the laser can act to desorp and ionize analytes.^1^, ^2^, ^3^, ^4^, ^5^, ^6^, ^7^, ^8^, ^9^, ^10^, ^11^, ^12^, ^13^, ^14^, ^15^, ^16^, ^17^, ^18^, ^19^, ^20^, ^21^, ^22^, ^23^, ^24^, ^25^, ^26^, ^27^, ^28^, ^29^, ^30^, ^31^, ^32^, ^33^, ^34^, ^35^, ^36^, ^37^, ^38^, ^39^, ^40^, ^41^, ^42^, ^43^, ^44^, ^45^ Indeed, the soft ionization laser method such as the original form of MALDI also belongs to this category.^1^ These techniques are not much different from MALDI regarding operational aspects, and, except for non‐occurring matrix‐related peaks, thus are basically applicable to imaging mass spectrometry.^32^, ^35^, ^37^, ^41^, ^43^, ^45^ In those cases, however, the challenges of MALDI imaging due to the matrix‐coating, i.e. the cumbersome experimental procedures and limitations in reproducibility and spatial resolution, are not solved by these techniques.

The techniques in the other category utilize planar solid substrates with fine structures on the uppermost surface layer as the target plate, so that the photon energies of the laser can reach analytes which are spread over and adsorbed onto the surface and achieve desorption and ionization of analytes.^20^, ^23^, ^46^, ^47^, ^48^, ^49^, ^50^, ^51^, ^52^, ^53^, ^54^, ^55^, ^56^, ^57^, ^58^, ^59^, ^60^, ^61^, ^62^, ^63^, ^64^, ^65^, ^66^, ^67^, ^68^, ^69^, ^70^, ^71^, ^72^, ^73^, ^74^, ^75^, ^76^, ^77^, ^78^, ^79^, ^80^ Desorption Ionization on Silicon (DIOS) is the representative technique in this category.^46^ This approach simplifies experimental procedures; hence these techniques are thought to be suited for automatic measurements using robotics. Practically, however, applications of these techniques to automate measurements are less advanced.

Porous alumina can be a device for achieving SALDI in the second category above as well as porous silicon.^68^ Among porous materials, through hole porous alumina membranes have a characteristic two‐dimensional (2D) ordered structure consisting of closely aligned straight through holes of sub‐micron in diameter and mass production of relatively wide sheets of the membranes is possible by industrial fabrication processes. We have undertaken a study aiming at the application of this through hole structure for SALDI. There are two reasons for taking particular note of the through hole structure. First, due to an increased degree of solution impregnation, it is expected that this structure can improve the reliability and reproducibility of desorption/ionization and extend the range of applicable sample solutions. Second, the permeation behavior of this structure is potentially useful for imaging mass spectrometry. All types of SALDI substrates including the porous alumina devices reported by Wada et al^68^ have the functional fine structures in only a shallow region of the substrate surface, and to the best of our knowledge there is no report concerning laser desorption/ionization assisted by a through hole porous substrate. Hence it is necessary to confirm experimentally that analytes are appropriately retained on the inner walls proximal to the substrate surface within the laser light action and the SALDI process is accomplished as well even by the through hole structure. The purpose of this study was to realize development of porous alumina substrates with the through hole structure retainable analytes and to prove them effective in generating gas‐phase ions of analytes. The feasibility study indicates that the structure can facilitate the ionization method and has several advantages over the conventional SALDI methods. We intentionally termed this novel ionization method, including its abbreviation; *Desorption Ionization Using Through Hole Alumina Membrane* (DIUTHAME).

## 2 EXPERIMENTAL

### 2.1 DIUTHAME chip

**Figure 1:**
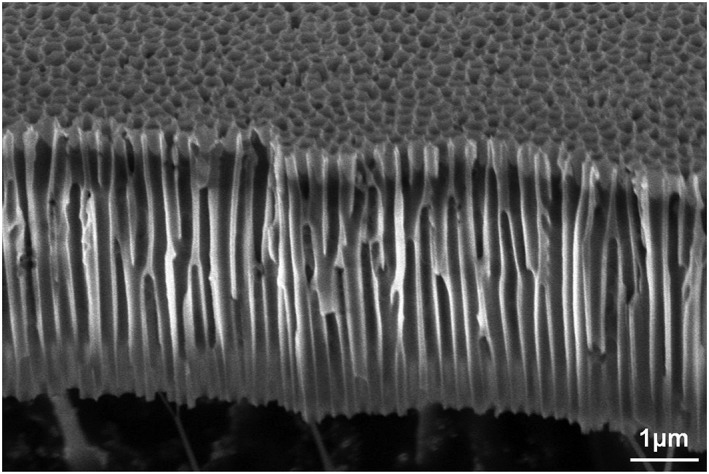
An example of the through hole porous alumina membranes observed by scanning electron microscopy (SEM). The scale bar indicates 1 μm

The through hole porous alumina membranes were fabricated by a wet anodization process from aluminum substrates. An aluminum (Al) plate (99.9%) was polished chemically at 80°C for 3 min in a solution of S‐CLEAN AL‐5000 (phosphoric acid and nitric acid) purchased from Sasaki Chemical Co., Ltd (Kyoto, Japan). The polished Al plate was anodized in 0.7 M malonic acid and then removed from the anodic oxide. The Al surface was formed of nano‐dimples corresponding to the shape of the bottom of the anodic oxide and a highly ordered anodic porous alumina was obtained by anodizing once again in 0.7 M malonic acid (two‐step anodizing). The anodic porous alumina membrane peeled off the Al plate. In this process, the action of self‐organizing dynamics creates the 2D ordered structure consisting of closely aligned straight through holes. The thickness of the membranes was adjusted by the post‐anodization process. The membrane was bonded to a titanium (Ti) frame by vacuum‐compatible epoxide adhesive. The obtained device was dipped in 1.7 M phosphoric acid solution to enlarge the pores, whose inner diameter and the open aperture ratio (OAR) of the structure can be controlled by the reaction time. Platinum (Pt) was coated at 10 nm on only the laser irradiation side by electron beam evaporation. Figure 1 shows an example of fabricated membranes observed by scanning electron microscopy (SEM).

In analogy with the role of fine structures in other SALDI methods, the size and shape of the through holes are thought to contribute to the conversion and transfer of photon energies. It has been confirmed by a separate preliminary experiment that the ionic yield depends to some extent on the inner diameter of the through holes. The hole diameter and OAR also have an effect on the mechanical strength of the membranes. Since the alumina membrane itself is mechanically fragile, a too weak membrane is difficult to deal with during fabrication and in use. The typical values for the hole diameter and OAR were set to 200 nm and 50%, respectively, considering the balance between the ion generating efficiency and the mechanical strength. In addition, the thickness of the membranes also has an effect on both the ion generating efficiency and the mechanical strength. While porous alumina becomes fragile in its thin form, the material acquires a mechanical flexibility to a small extent and can bend slightly with maintaining restorability until its breaking point. The typical value for the membrane thickness was set to 5 μm considering the balance between the ion generating efficiency and the mechanical strength.

It has been indicated from a preliminary experiment that a metal coating on the membrane surface is essential. Because bulk alumina is an insulating material itself, a conductive coating is important to determine a certain electric potential for the membrane surface. On the other hand, the high hydrophilic property of the alumina surface is lost by a metal coating because the hydrophilicity of the metal surface is generally lower than that of alumina. The hydrophobic behavior is further emphasized by a liquid‐repellent action due to the surface figure of the through hole membrane. This fact affects impregnation of a sample solution into the through holes. From a preliminary experiment comparing (i) the membrane coated on both sides, (ii) the membrane coated on a single side that was exposed to the laser, and (iii) the membrane coated on a single side then the opposite side exposed to the laser, it was decided to use structure (ii). It has been empirically confirmed that various kinds of metal are effective as the coating material. Pt was selected as the coating material considering its chemical stability and the mechanical strength to reinforce the membrane.

**Figure 2:**
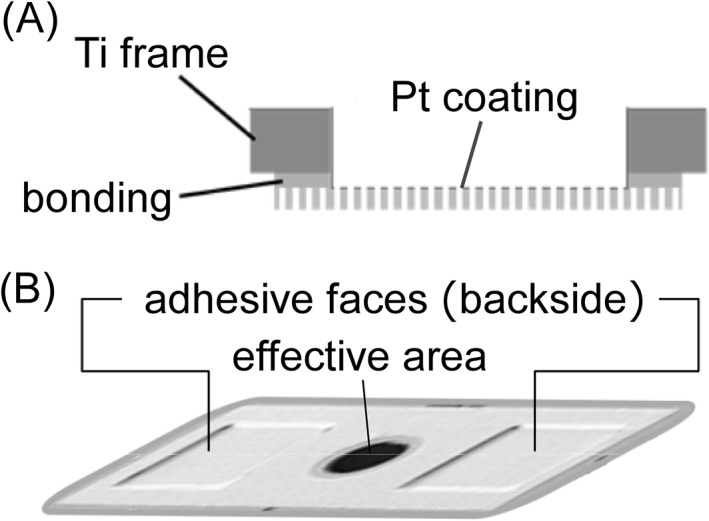
Schematic view A, and photograph B, of the DIUTHAME chip in its final form

In order to ease handling in practice, the membrane was bonded to a Ti plate with Ø3 mm aperture, in which the exposed part of the membrane became the effective area (Figure 2). The Ti plate not only keeps the device form as a frame, but also provides an electrical contact to the Pt coating surface. As the final form of the device, the Ti plate has adhesive faces on the backside (viewed from the mass analyzer) to mount on a target plate. Here we will call this device a DIUTHAME chip.

### 2.2 Evaluation experiment

Angiotensin II (human), water (LC/MS Ultra CHROMASOLV) and acetonitrile (biotech grade, ≥99.93%) were purchased from Sigma‐Aldrich Co. (St Louis, MO, USA); polyethylene glycol (PEG2000), citric acid monohydrate (≥ 99.5%), diammonium hydrogen citrate (≥ 99%) and acetone (≥ 99.5%) were purchased from Kanto Chemical Co., Inc. (Tokyo, Japan); trifluoroacetic acid (TFA; 25% in water) was purchased from Merck KGaA (Darmstadt, Germany); formic acid (≥98%) was purchased from Wako Pure Chemical Industries, Ltd (Osaka, Japan); and the peptide mixture (Peptide Calibration Standard II) was purchased from Bruker Daltoniks GmbH (Bremen, Germany).

Angiotensin II (1.0 mg) was dissolved in water (1000 μL) to make a 1mM aqueous solution. An aqueous solution of the peptide mixture was made from Bruker Peptide Calibration Standard II; dissolving the contents of the tube in 12.5 μL water produced a ten times higher concentration than that of the recommended procedure described in the instructions for use. Citric acid and diammonium hydrogen citrate were dissolved in water individually to make 0.2 M aqueous solutions. PEG2000 (5.0 mg) was dissolved in 1000 μL acetone. The aqueous solution of angiotensin II was diluted and then mixed with equal volumes of aqueous solutions of citric acid and diammonium hydrogen citrate and acetonitrile. The aqueous solution of the peptide mixture was mixed with equal volumes of aqueous solutions of citric acid and diammonium hydrogen citrate.

**Figure 3:**
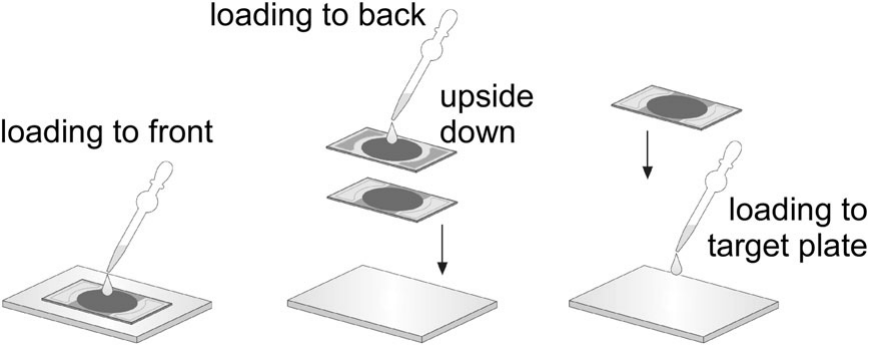
Protocols for loading sample solution to the DIUTHAME chip. A sample solution can be applied by either (i) loading onto the Pt coating surface of the DIUTHAME chip mounted on a target plate, or (ii) loading onto the hydrophilic surface of the DIUTHAME chip placed upside down then mounting the chip on a target plate in the right manner, or (iii) loading onto a target plate followed by mounting the DIUTHAME chip so that the droplet is covered with the effective area, depending on the solvent composition

Three protocols for loading sample solution to the DIUTHAME chip are possible (Figure 3); (i) loading a small volume (1–2 μL) of sample solution onto the Pt coating surface of the DIUTHAME chip mounted on a target plate; (ii) loading a small volume (1–2 μL) of sample solution onto the hydrophilic surface of the DIUTHAME chip placed upside down, then mounting the chip on a target plate after impregnating with the solution; (iii) loading a small volume (1–2 μL) of sample solution onto a target plate followed by mounting the DIUTHAME chip so that the droplet is covered with the effective area. Every protocol was tested for the case of angiotensin II. The peptide mixture and PEG2000 were loaded by protocols (i) and (ii) and protocol (i), respectively.

Positive mode mass spectra were acquired by a MALDI time‐of‐flight mass spectrometer (Bruker Ultraflex II TOF/TOF) equipped with a nitrogen laser (wavelength: 337 nm).

## 3 RESULTS AND DISCUSSION

### 3.1 Measurement of a single peptide

When the sample solution was loaded onto the front side (Pt coating surface) of the DIUTHAME chip, a swollen round droplet formed on the surface and a longer time was needed for impregnation than the case of loading from the opposite side. When the sample droplet on the target plate was covered by the DIUTHAME chip this delivered a state of capillary adhesion which was caused by the droplet being squeezed between the target plate and the membrane, while the surplus fluid came out of the effective area and spread over the whole backside. Nevertheless, it was possible to obtain mass spectra of angiotensin II for every case and no notable difference was observed in the resultant spectra. Because the conventional SALDI plates have a very high hydrophobicity, improper loading of samples with such a water content often causes failure of measurements in our experience. In contrast, the improved usability of the DIUTHAME chip where users do not have to worry about the solvent composition should be noted.

As an apparent point of difference from MALDI, the so‐called “sweet spot” does not exist with DIUTHAME. Although inhomogeneous impregnation of the sample solution into the through holes to some extent was caused by excess volume and/or situation (e.g. air bubbling) of the loaded sample solution, almost the same mass spectra were observed at any point on the effective area except for the inhomogeneous parts. Consequently, the reproducibility of mass spectra obtained by laser irradiation to the target in a random manner was dramatically improved in comparison with that of MALDI. Furthermore, good reproducibility was also observed between different targets.

The laser intensity for acquiring good mass spectra was substantially higher than that for MALDI. This tendency is common among other SALDI methods such as DIOS. While the conventional SALDI methods based on fine structures of the uppermost surface layer require careful tuning of the laser intensity due to its narrow working range, DIUTHAME shows a relatively modest dependency upon the laser intensity and provides an easier adjustment than conventional SALDI.

In general MALDI experiments, consecutive mass spectra obtained from an identical sweet spot are accumulated for several (~100) laser shots. Observation of a real‐time DIUTHAME mass spectrum in the course of accumulating at an identical exposure spot indicated that prominent cumulative peak intensities were found in the initial few laser shots and the peak intensity dropped suddenly in the subsequent laser shots. This behavior did not change significantly by raising/lowering the laser intensity. Because it was not beneficial from the view point of signal‐to‐noise ratio (S/N) even though extending the laser irradiation period, the number of laser shots for each spot was set to 20 shots. Under this laser condition irradiation marks clearly observable by the video camera of the mass spectrometer remained on the membrane surface. SEM observation of the irradiation marks revealed that the ordered structure consisting of through holes was maintained without breakage.

**Figure 4:**
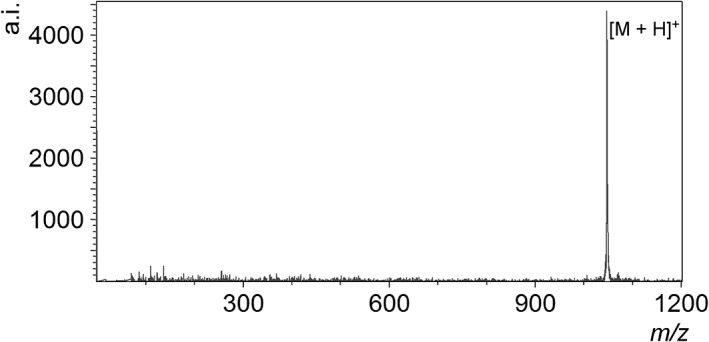
Mass spectrum obtained from an aqueous solution of angiotensin II (human) by using DIUTHAME. The concentration of angiotensin II in the aqueous solution was set to 1 μM; this solution was then mixed with equal volumes of 0.2 M aqueous citric acid solution, 0.2 M aqueous diammonium hydrogen citrate solution, and acetonitrile. The final concentration of angiotensin II and water content in the solvent composition were 0.25 μM and 75%, respectively. The sample solution was loaded to the DIUTHAME chip by protocol (iii) described in the text

Figure 4 shows an example of a DIUTHAME mass spectrum obtained from an aqueous solution of angiotensin II. Although the spectrum is similar to the MALDI spectrum and dominated by a [M + H]^+^ peak, this good spectrum was acquired without the matrix suppression function of the mass spectrometer since intense signal components in the low‐*m/z* region are absent, unlike MALDI. The present result contains many weak peaks in the low‐*m/z* region; however, these peaks have been found in a blank control spectrum obtained from the aqueous solution without angiotensin II. Besides, a bare DIUTHAME chip without loading sample has delivered only Pt‐related peaks occurring from the coating material in accord with increased laser intensity. Therefore, these peaks that appear in the low‐*m/z* region are presumably attributed to impurities in the solvent but not to fragment ions of the peptide. Because of the absence of intense matrix signals, DIUTHAME is thought to be sensitive for low amounts of impurities. The DIUTHAME chip itself has an acceptable level of cleanliness in practice and does not generate notable noise components except for Pt‐related species including their cluster ions. This property was retained for DIUTHAME chips preserved long term (>6 months) in a desiccator.

Sequence‐related fragment ions of the peptide have not been found by thoroughly investigating even higher *m/z* regions of the DIUTHAME spectra recorded in the reflector mode. While metastable decay products of the peptide are generated in MALDI according to increased laser intensity and correspond to low‐energy collision‐induced dissociation (CID) products such as b‐ and y‐series ions, the DIUTHAME spectra have been dominated by the [M + H]^+^ peak and not shown peaks clearly attributable to metastable decay product ions for a certain range of the laser intensity. This fact should not readily lead to the conclusion that DIUTHAME has a softness equal to or more than MALDI; however, it implies that there is no process (or situation) causing metastable decay in DIUTHAME. A model has been proposed for metastable decay process in MALDI; generated ions passing through a dense plume are subjected to multiple energetic collisions, which bring about collisional activation and consequent metastable decay patterns similar to low‐energy CID.^82^ In contrast, it is suggested for DIUTHAME that laser irradiations may not generate a dense plume at all, or the thermodynamic property of the plume may be quite different from that of MALDI.

A mixture of aqueous solutions of the peptide, 0.2 M citric acid, 0.2 M diammonium hydrogen citrate and acetonitrile in equal volumes was employed in the measurement shown in Figure 4. This preparation procedure is commonly required for other SALDI methods such as DIOS. A sample solution with the peptide simply dissolved in water was measured by DIUTHAME and delivered only a faint [M + Na]^+^ peak. The same tendency has been observed for other peptide samples. Although citric acid is thought to be the proton donor, a modified preparation procedure in which the combination of citric acid and diammonium hydrogen citrate was replaced by TFA or formic acid failed to effectively detect [M + H]^+^ ions and brought increased noise levels in the low‐*m/z* region.

The detection limit of DIUTHAME for the aqueous solution of angiotensin II was 0.1 μM so far. Peptide molecules larger than insulin (MW: 5733) have not been detected by DIUTHAME yet.

### 3.2 Measurement of the peptide mixture

**Figure 5:**
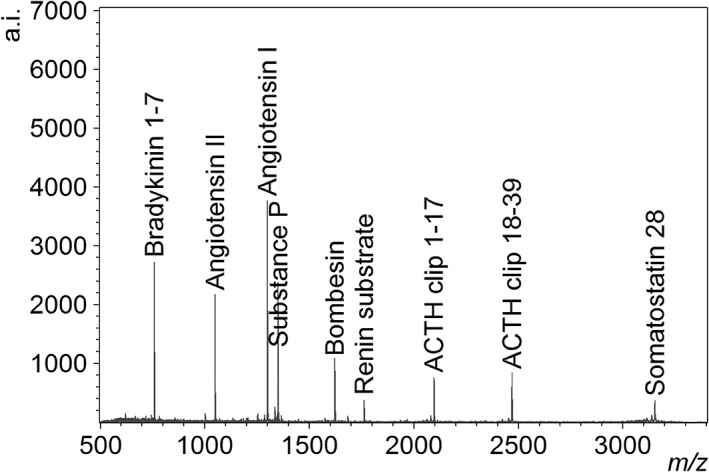
Mass spectrum obtained from an aqueous solution of the peptide mixture (Bruker Peptide Calibration Standard II) by using DIUTHAME. The concentration of the peptide contents in the aqueous solution was set to ten times higher than the standard preparation in the instructions provided by the manufacturer; this solution was then mixed with equal volumes of 0.2 M aqueous citric acid solution, 0.2 M aqueous diammonium hydrogen citrate solution. The sample solution was loaded to the DIUTHAME chip by protocol (ii) described in the text

Figure 5 shows a DIUTHAME mass spectrum obtained from the aqueous solution of nine kinds of mixed peptides. All peptide contents were observed as [M + H]^+^ peaks; however, the peak intensities reduced in a high *m/z* side compared with a typical MALDI spectrum obtained from this peptide mixture. In addition, a certain peptide peak (renin substrate) tended to be weak. This phenomenon seems to be a kind of ion suppression but has not been elucidated.

Unlike the case of a single peptide, the measurement of the peptide mixture depended on loading protocols and the best result was obtained by loading the sample solution directly onto the hydrophilic surface of the DIUTHAME chip. In that case, equally good spectra were obtained whatever the composition of the organic solvent in the solvent composition, thus an aqueous solvent was preferable from a view point of pipetting work.

### 3.3 Measurement of polyethylene glycol

**Figure 6:**
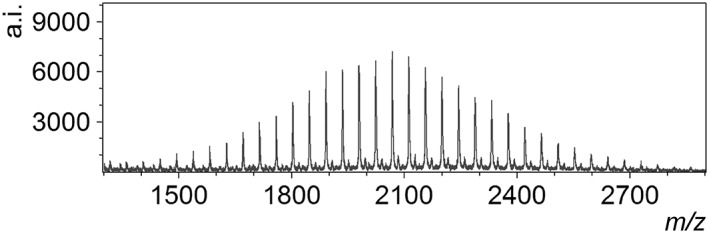
Mass spectrum obtained from polyethylene glycol (PEG2000) dissolved in acetone by using DIUTHAME. The concentration of PEG2000 was 5 mg/mL. The sample solution was loaded to the DIUTHAME chip by protocol (i) described in the text

Figure 6 shows a DIUTHAME mass spectrum obtained from PEG2000 dissolved in acetone. [M + Na]^+^ peaks with the envelope reflecting the molecular weight distribution of PEG2000 were clearly observed. [M + Na]^+^ ions were detected without a cationizing agent such as NaTFA. Furthermore, the ion signals generated from an identical exposure spot did not decrease after numerous laser shots, even showing a tendency to increase, in contrast to the cases of peptide measurements. Due to the persistent signals at a single exposure spot (~1000 shots), a mass spectrum superior to the case of MALDI using general matrices was acquired.

The reason for the excellent performance of DIUTHAME for polyethylene glycol is thought to be as follows. Assuming [M + Na]^+^ to be the preformed ions, the through hole porous alumina membranes fabricated in the wet process may provide a sodium‐rich environment suitable for formation of the cationized molecules. Besides, acetone as the solvent for polyethylene glycol may facilitate spreading and adsorption of the molecules on the inner walls. Although polyethylene glycol is known as a relatively feasible compound in other SALDI methods also, the high persistence of the ion signals is a characteristic feature of DIUTHAME and not found in other SALDI methods. Because an exposed spot maintains the ordered structure consisting of through holes as mentioned above, it is suggested that desorption of the preformed ions persists after removal of the uppermost layer of the membrane.

The other important feature which is thought to be a benefit of the through holes is the tolerance for a dense sample. While other SALDI methods require a rigorous adjustment of sample concentration because the ionizing function is lost by a dense sample masking the surface structure, DIUTHAME allows an extended sample condition toward a higher concentration due to the through holes playing the role of drains. This can be beneficial in practical use.

## 4 CONCLUSIONS

This study verified for the first time the concept that a through hole porous alumina membrane can be used as a SALDI substrate. Particularly, it was found that the membrane remains effective for SALDI with through holes of the aspect ratio (the membrane thickness to the hole diameter) exceeds 20 which is much larger than those in previous cases, the typical value of the aspect ratio (the hole depth to the hole diameter), for instance, was set to 5 in the porous alumina substrate reported by Wada et al. The high aspect ratio not leading to disruption of SALDI process is a discovery reversing the preconceived idea about SALDI substrate. The through hole porous alumina membranes have been proven to have equivalent functionality with other types of SALDI plates in terms of conversion and transfer of laser photon energies achieving desorption and ionization of analytes. Additionally, it has been confirmed that the capillary action of the through holes with very high aspect ratio enables the loading protocol in which sample solutions are applied to the hydrophilic surface opposite to the exposed side. Furthermore, in comparison with the conventional SALDI plates, the following benefits from the through hole structure have been found; (i) Users do not have to worry about the solvent composition, because aqueous solutions can be applied easily. (ii) A wider working range of the laser intensity provides an easier adjustment. (iii) The tolerance level of sample concentration extends toward a higher side due to the through holes acting as drains. (iv) An extremely high persistence of signals generated from a single exposure spot is achieved for some samples such as polyethylene glycol dissolved in acetone. We propose this laser desorption/ionization method employing the through hole porous alumina membranes, DIUTHAME, as a novel ionization method which greatly improves the usefulness of SALDI.

Finally, and moreover, a possibility becomes explicit from this 2D ordered structure consisting of closely aligned straight through holes and the fact that analytes impregnated from the opposite side of the surface exposed to laser have been detected; DIUTHAME can be used for imaging mass spectrometry, which is impossible with the conventional SALDI plates. Intensive studies for exploring the feasibility of DIUTHAME in imaging mass spectrometry are currently underway.^83^
